# Hetero-modification of TRAIL trimer for improved drug delivery and *in vivo* antitumor activities

**DOI:** 10.1038/srep14872

**Published:** 2015-10-08

**Authors:** Li-Qiang Pan, Wen-Bin Zhao, Jun Lai, Ding Ding, Xiao-Yue Wei, Yang-Yang Li, Wen-Hui Liu, Xiao-Yue Yang, Ying-Chun Xu, Shu-Qing Chen

**Affiliations:** 1 Institute of Drug metabolism and Drug analysis, College of Pharmaceutical Sciences, Zhejiang University, Hangzhou, 310058, China; 2 Zhejiang Hisun Pharmaceutical Inc, 46 Waisha Road, Jiaojiang District, 317000 Taizhou, China

## Abstract

Poor pharmacokinetics and resistance within some tumor cell lines have been the major obstacles during the preclinical or clinical application of TRAIL (tumor-necrosis-factor (TNF)-related apoptosis-inducing ligand). The half-life of TRAIL_114-281_ (114 to 281 amino acids) was revealed to be no more than 30 minutes across species. Therefore maleimido activated PEG (polyethylene glycol) and MMAE (Monomethyl Auristatin E) were applied to site-specifically conjugate with the mutated cysteines from different monomers of TRAIL successively, taking advantage of steric effects involved within TRAIL mutant conjugations. As a result, TRAIL trimer was hetero-modified for different purposes. And the resulting PEG-TRAIL-vcMMAE conjugate exhibited dramatically improved half-life (11.54 h), favourable *in vivo* targeting capability and antitumor activities while no sign of toxicity in xenograft models, suggesting it’s a viable therapeutic and drug delivery strategy.

After binding with death receptors (DR4 and DR5), homotrimeric tumor-necrosis-factor (TNF)-related apoptosis-inducing ligand (TRAIL) could induce apoptosis in various cancer cells instead of most normal human cell types, followed with the internalization of ligand-receptor complex by tumor cells^1^,^2^,^3^. To overcome TRAIL resistance resulted from the defects of intracellular apoptotic pathway in some tumor cells, the internalization process has been utilized to deliver highly toxic chemical (Monomethyl Auristatin E, MMAE) into cytoplasm via TRAIL-MMAE conjugates^4^,^5^,^6^,^7^,^8^,^9^,^10^. Therefore, the released MMAE could inhibit tumor cell division by blocking the polymerization of tubulin, after the enzymatic hydrolysis of conjugates in lysosome^11^. However, poor pharmacokinetics has been the major obstacle during the preclinical or clinical application of TRAIL, and half-life of TRAIL ranges from ~3 min to 5 min in rodents and ~23 min to 30 min in nonhuman primates^12^. In this way, half-life of TRAIL-MMAE conjugates doesn’t seem to benefit much from the conjugation with MMAE because of its similar molecular weight to TRAIL, and needs to be prolonged to achieve better bioavailability for ultimate antitumor activity, before eliminated by glomerular filtration.

PEGylation offers a possibility to address the requirements of TRAIL pharmacokinetics, since polyethylene glycol (PEG) has been successfully applied to the improvement of many protein drugs, and is non-toxic, non-immunogenic, and highly soluble in water^13^. Hence the co-modification of TRAIL trimer with PEG and vcMMAE (maleimidocaproyl-valine-citrulline-MMAE) provides a possible way to combine the favorite properties of PEG and MMAE. But how to PEGylate TRAIL-vcMMAE conjugates efficiently remains to be a tough problem. N-terminal PEGylation of TRAIL could merely maintain no more than 10% antitumor activity of native TRAIL, which would hinder the antitumor activity of TRAIL-vcMMAE in spite of limited half-life elongation^14^. Homotrimeric TRAIL has a unique three-dimensional structure, a zinc chelated by Cys230 from three TRAIL monomers to stabilize the tertiary structure^15^. Asn-109, a potential N-linked glycosylation site within TRAIL_95-281_, thus was mutated to cysteine to facilitate the conjugation of TRAIL with maleimido modified MMAE^16^. Another TRAIL PEGylation strategy also took advantage of the rapid reaction between mutated Cys109 and maleimide, namely TRAIL mutant N109C was conjugated with methoxy-PEG-maleimide (mPEG-MAL), and has achieved better *in vivo* activity than native TRAIL^14^. Interestingly, PEGylated TRAIL mutant N109C trimer left one monomer unmodified according to the previous MALDI-TOF-MS (Matrix-Assisted Laser Desorption/Ionization Time of Flight Mass Spectrometry) result, which was probably aroused due to steric effects. Because PEGylation at N-terminus of TRAIL obtained tri-PEGylated trimer. Consequently, we could make full use of steric effects to co-modify TRAIL trimer with PEG (the larger) and MMAE (the smaller) successively at the same site of different monomers (Fig. 1). Herein, we describe the design, synthesis, and application of PEG-TRAIL-vcMMAE (PEG-N109C-vcMMAE) conjugates for the efficient inhibition of death receptors (DR4 and DR5) positive tumors.

## Results

### Characterization of PEG-TRAIL-vcMMAE

**Figure 2:**
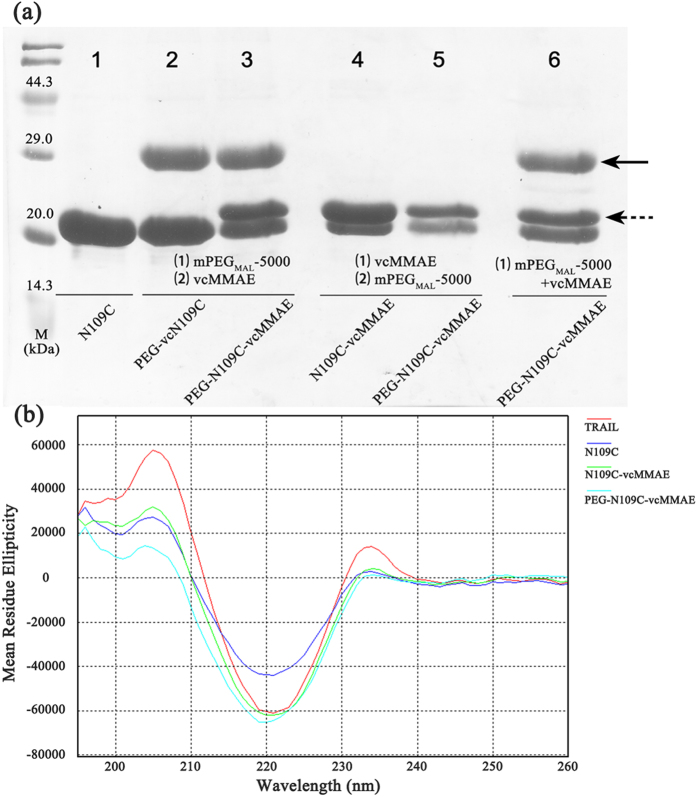
Characterization of PEG-TRAIL-vcMMAE conjugates. (**a**) SDS-PAGE analysis of N109C and the outcome of different modification steps. N109C was conjugated with PEG or MMAE for 40 min after one hour reduction by TCEP (tris (2-carboxyethyl) phosphine). Serial number in bracket (e.g., (1)) indicates the modification order. Lane 6 represents the modification step that PEG was added 30 seconds ahead of MMAE. The black arrow and dashed arrow indicate PEG-N109C and N109C-vcMMAE monomer, respectively. (**b**) Comparing the secondary structure of TRAIL_95-281_, N109C, N109C-vcMMAE and PEG-N109C-vcMMAE via Circular dichroism (CD) spectroscopy.

Methoxy-PEG maleimide sized about 5000 Da was chosen to modify N109C efficiently, as larger PEG might be unable to reach the mutated cysteine of N109C because of steric effects, while smaller PEG could not contribute to TRAIL pharmacokinetics significantly. Effect of reaction order on yield during the co-modification were evaluated by SDS-PAGE. As shown in Fig. 2a, when N109C reacted with PEG first, followed by the reaction with MMAE, the PEG-N109C-vcMMAE could be successfully synthesized with a monomer modification ratio of about 1:1:1 (PEG modified monomer: MMAE modified monomer: unmodified monomer). In contrast, when N109C-vcMMAE conjugates were first prepared, PEG failed to further modify the conjugates (Lane 5). Besides, although PEG was added 30 seconds ahead of MMAE, the co-modification of N109C with PEG and MMAE still succeed despite of the overall decrease in yield (Lane 6). Therefore, the PEG-TRAIL-vcMMAE conjugates could be obtained via steric effects involved in the co-modification of TRAIL with PEG and MMAE. The conjugation of N109C with MMAE was further confirmed by Waters Xevo® G2-S Q-TOF (Supplementary Figure S1). And size-exclusion chromatography (SEC) demonstrated that N109C was a mixture of trimer and dimer, while N109C-vcMMAE and PEG-N109C-vcMMAE was trimer as expected (Supplementary Figure S2). N109C dimer resulted from wrong intramolecular disulfide bond formed within mutated cysteines. The SEC results implied the modification with MMAE or co-modification with PEG and MMAE at mutated cysteine would not change TRAIL tertiary structure.

TRAIL maintained its secondary structure composition (mainly beta-sheet, featured by absorbance near 220 nm) after the conjugation with PEG and MMAE (Fig. 2b). Comparing with N109C, the main secondary structure (β-sheet content) of PEG-N109C-vcMMAE is much closer to N109C-vcMMAE and native TRAIL. These results indicate the secondary structure could be restored after reduction and co-modification of N109C, although the Asn-109 to Cys mutation altered the main secondary structure of TRAIL to some extent.

### Apoptotic mechanism of PEG-TRAIL-vcMMAE conjugates

**Figure 3:**
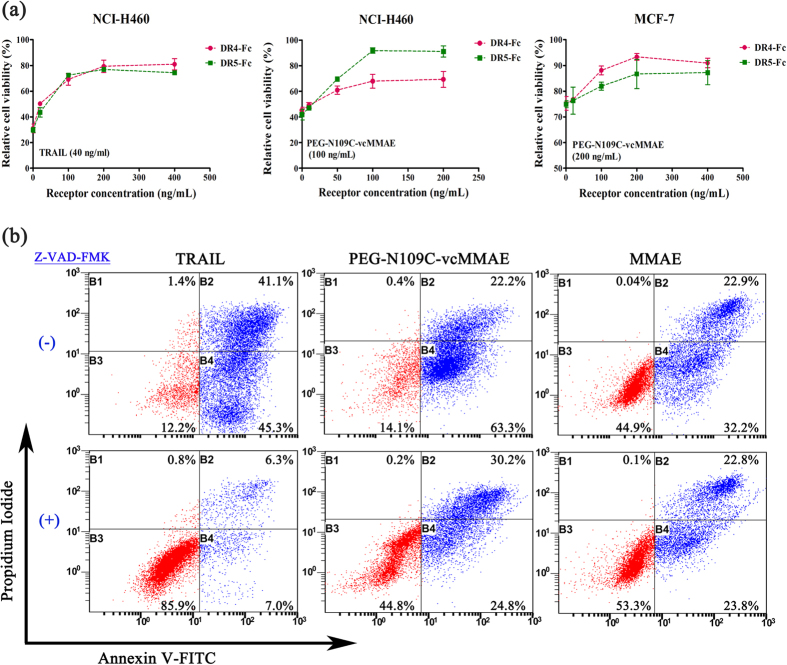
Apoptotic mechanism of PEG-TRAIL-vcMMAE conjugates. (**a**) Competitive binding of TRAIL or PEG-N109C-vcMMAE with DR4-Fc/DR5-Fc to confirm the internalization pattern of conjugates. Receptor concentration refers to the concentration of DR4 or DR5. The results were expressed as mean ± S.D. (n = 3) (**b**) Inhibition of TRAIL extrinsic apoptotic pathway *via* the addition of pan-caspase inhibitor Z-VAD-FMN. Early (B4 gate) and late stage (B2 gate) apoptotic tumor cells were designated in green.

Since TRAIL was supposed to deliver MMAE into tumor cell cytoplasm via DR-mediated internalization, specific blockage of this process by DR4-Fc/DR5-Fc was applied to confirm the TRAIL/DR interaction. As shown in Fig. 3a, both DRs could specifically inhibit TRAIL or PEG-TRAIL-vcMMAE conjugates induced apoptosis, and showed a dose-dependent relationship in two type tumor cells. Besides, DR5 was superior to DR4 in inhibiting conjugates-induced apoptosis upon NCI-H460 cells, while they functioned equally in the inhibition of TRAIL-induced apoptosis.

To find out the contribution of the payload (MMAE) in apoptosis-inducing activity of PEG-TRAIL-vcMMAE, the extrinsic TRAIL pathway was blocked through the co-treatment of a pan-caspase inhibitor (Z-VAD-FMK). In Fig. 3b, TRAIL induced apoptosis (early and late stage) in NCI-H460 cells was nearly completely blocked in the presence of Z-VAD-FMN (from 86.4% to 13.3%). However, only 30.5% was blocked in the case of PEG-N109C-vcMMAE induced apoptosis (from 85.5% to 55.0%), suggesting that MMAE plays a major role in killing tumor cells.

### Biological activities of PEG-TRAIL-vcMMAE

**Figure 4:**
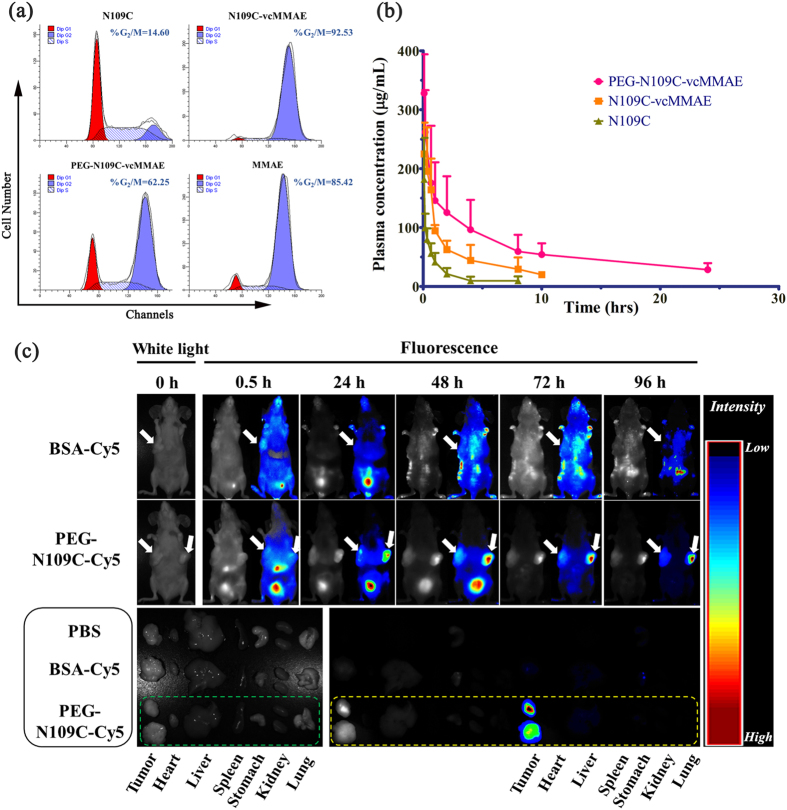
Biological activities of PEG-TRAIL-vcMMAE (**a**) Selective induction of G_2_ growth arrest. MCF-7 cells were exposed to 1 μM N109C and its conjugates or 0.5 μM MMAE for 8 hrs before analysis by flow cytometry. Blue peak designates the position of G_2_-M phase populations. %G_2_/M indicates the percentage of cells arrested at the G_2_-M phase. (**b**) Pharmacokinetic profiles of N109C and its conjugates. Sprague-Dawley (SD) rats were administered an i.v. injection of N109C, N109C-vcMMAE or PEG-N109C-vcMMAE (1 mg per rat), and their concentrations in blood were determined by ELISA. Results are expressed as mean ± S.D. from 4 different rats (n = 4). (**c**) *In vivo* distribution of PEG-N109C-Cy5. Tumor locations were designated by white arrows. Both flanks of PEG-N109C-Cy5 group mouse were inoculated with NCI-H460 cells.

As MMAE could inhibit tumor cell division by blocking the polymerization of tubulin, thus we examined the MCF-7 cells treated with TRAIL and its conjugates for the cell cycle distribution. As depicted in Fig. 4a, 14.60% of TRAIL treated MCF-7 cells located in the G_2_-M phase, indicating different mechanism involved in its apoptotic activity instead of cell cycle arrest. On the other hand, N109C-vcMMAE (92.53%) induced the most cell cycle arrest in MCF-7 cells followed by PEG-N109C-vcMMAE (62.35%). Positive control MMAE also inhibited 85.42% of MCF-7 cells from further division even at a half dose (0.5 μM) of other MMAE conjugates (1 μM). The cell cycle arrest results demonstrated the conjugation with MMAE conferred the capability of G_2_-M phase arrest to N109C-vcMMAE and PEG-N109C-vcMMAE on TRAIL-resistant MCF-7 cells (Fig. 4a). And according to the *in vitro* antitumor activity results (Supplementary Figure S3), PEG-N109C-vcMMAE was revealed attenuated antitumor activities derived from the common fault of PEGylation.

We also studied the pharmacokinetics of PEG-N109C-vcMMAE and N109C-vcMMAE, and found out the half-life of PEG-N109C-vcMMAE in rat was elongated to 11.54 h in comparison with N109C (1.91 h) and N109C-vcMMAE (4.26 h) (Table 1). Thus the bioavailability and half-life of TRAIL have been improved dramatically through the co-modification with PEG and MMAE (Fig. 4b and Table 1).

### Evaluation of the *in vivo* tumor targeting capability of PEG-TRAIL-vcMMAE

To assess the *in vivo* targeting effect of PEG-N109C-vcMMAE, sulfo-cyanine5-maleimide (water-soluble Cy5, a near- IR fluorophore) was conjugated to PEG-N109C to obtain PEG-N109C-C  g/mol), Cy5-maleimide could be an ideal fluorescent marker without deviating from structural effect of conjugating PEG-N109C with MMAE; thus the application of PEG-N109C-Cy5 would veritably reflect the *in vivo* distribution of PEG-N109C-vcMMAE. On the other hand, BSA (bovine serum albumin) was modified with Cy5 likewise as non-binding control, for its similar molecular weight (66 kDa) with N109C trimer (67 kDa). After intravenous administration of PEG-N109C-Cy5 and BSA-Cy5 in NCI-H460 lung cancer mouse xenograft model, the fluorescence was monitored using Maestro *in vivo* imaging system (CRi, USA) at different time points. At 0.5 h, both PEG-N109C-Cy5 and BSA-Cy5 were found to be systemically distributed, and part of them were observed in bladder owing to glomerular filtration (Fig. 4c, the upper half). More and more PEG-N109C-Cy5 conjugates accumulated in tumor from 24 h, and all of the conjugates were metabolized at 96 h, except the accumulation in two tumor sites (arrowed). But the distribution of BSA-Cy5 seems to be in a random pattern across the whole experiment. The specific accumulation of PEG-N109C-Cy5 in the tumor was further confirmed according to the spectral fluorescence images of the dissected organs (tumor, heart, liver, spleen, stomach, and kidney) (Fig. 4c, the bottom half). These results indicate PEG-N109C-vcMMAE could specifically deliver MMAE to target tumor cells, and the non-binding control showed the enhanced-permeability-and-retention effect (EPR) was not the major reason for the specific accumulation of PEG-N109C-Cy5. And PEG-TRAIL has great potential to be an active tumor targeting strategy for delivering highly toxic molecules.

### *In vivo* antitumor activities of TRAIL and its conjugates

**Figure 5:**
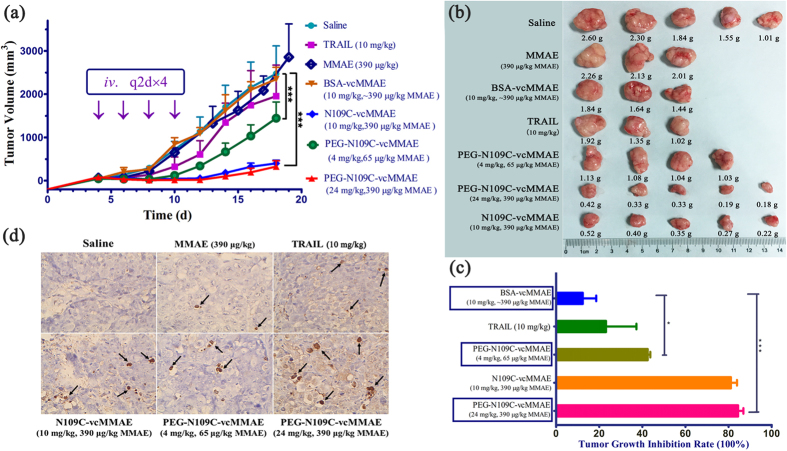
*In vivo* antitumor activities of PEG-TRAIL-vcMMAE. (**a**) *In vivo* antitumor activities of TRAIL and its conjugates. When tumor volume reached ~50 mm^3^, all the samples were administered intravenously once every two days for 4 times. Tumor volume was calculated by the formula: *V* *=* *L* × *W*^2^/2, *L* and *W* refer to longitudinal and transverse tumor diameters respectively. ***P < 0.001 (two-way analysis of variance, ANOVA). (**b**) Dissected tumor from different experimental groups. (**c**) Tumor growth inhibition rate was calculated according to the tumor weight. *P < 0.05 and ***P < 0.001 (one-way analysis of variance, ANOVA). (**d**) TUNEL analysis of histological sections from different groups. Representative apoptotic cells were arrowed. Magnification 400×.

*In vivo* antitumor activities of N109C-vcMMAE and PEG-N109C-vcMMAE were studied on NCI-H460 lung cancer mouse xenograft model. BSA-vcMMAE was used as non-binding control. TRAIL-sensitive tumor cells could acquire resistance to TRAIL-induced apoptosis after repeated application of high doses of TRAIL^17^,^18^,^19^. For example, NCI-H460 cells are highly TRAIL-sensitive with high-expression of cell-surface DR4 and DR5, but a subpopulation of parental NCI-H460 cells begun to overgrow after discontinuance of daily application of TRAIL or PEG-TRAIL *in vivo*^14^. Therefore, NCI-H460 cells were chosen to facilitate the comparison of *in vivo* antitumor activities of TRAIL and PEG-N109C-vcMMAE. The dosages of N109C-vcMMAE and PEG-N109C-vcMMAE were based on the MMAE loading, since the antitumor activities of PEG-N109C-vcMMAE mainly depended on the cytostatic activity of MMAE (Fig. 3b). As depicted in Fig. 5a, PEG-N109C-vcMMAE at 24 mg/kg (0.39 mg/kg MMAE) showed the best *in vivo* antitumor activities, followed by N109C-vcMMAE at 10 mg/kg (0.39 mg/kg MMAE). And low-dose PEG-N109C-vcMMAE (4 mg/kg) was also capable of killing tumor cells significantly. In contrast, TRAIL (10 mg/kg) failed to inhibit tumor growth efficiently when dosed once every two days for four times. Intriguingly, MMAE, as a highly toxic agent, was shown subtle difference from saline group in tumor inhibition *in vivo*, indicating the importance of TRAIL delivery. BSA-vcMMAE also exhibited little antitumor activity in comparison with saline, which is consistent with the results of *in vivo* distribution of BSA-Cy5. The *in vivo* antitumor effect of N109C-vcMMAE was superior to TRAIL, suggesting the conjugation with MMAE could favor TRAIL with improved activity on TRAIL-resistant subpopulation of NCI-H460 cells. And the co-modification of TRAIL with PEG and MMAE further elevated the tumor inhibition activities of TRAIL, as a result of better bioavailability and overcoming TRAIL resistance within tumor cells. To confirm the *in vivo* antitumor activities of TRAIL and its conjugates, the dissected tumor from xenograft mouse were examined and weighed, and the tumor inhibition rate based on tumor weigh was calculated (Fig. 5b,c).

To examine the apoptosis inside tumor, TUNEL (Terminal deoxynucleotidyl transferase dUTP nick end labeling) was applied to detect DNA fragmentation that results from apoptotic signaling cascades. PEG-N109C-vcMMAE, N109C-vcMMAE and TRAIL induced apoptosis in tumor cells obviously, while MMAE alone could seldom kill tumor cells *in vivo* (Fig. 5d and Supplementary Figure S4). According to these results, tumor cells probably obtained resistance to TRAIL after several TRAIL dosages, and begun to overgrow ever since. The results above are consistent with the *in vivo* antitumor activities of TRAIL and TRAIL conjugates (Fig. 5a–c).

### Toxicity studies of PEG-TRAIL-vcMMAE

**Figure 6:**
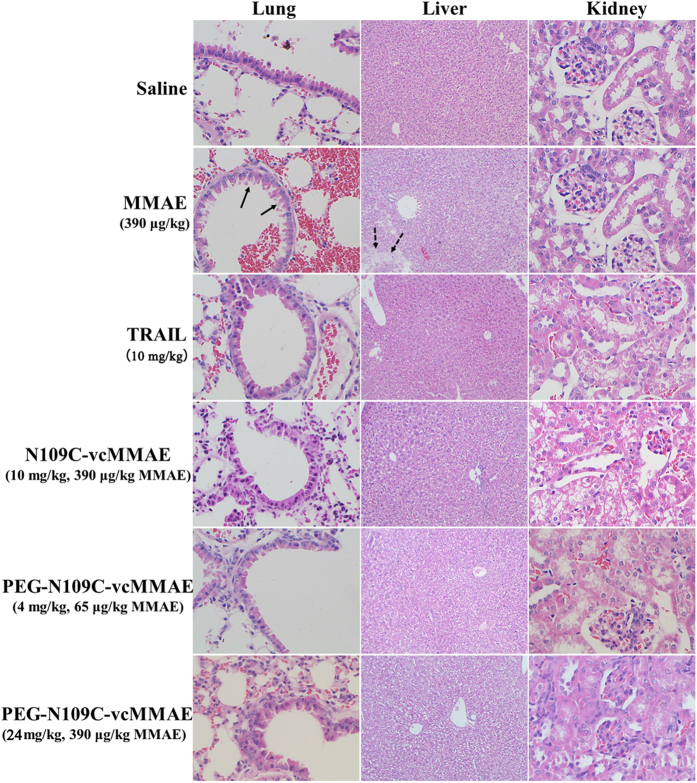
Histologic investigations of lungs, livers and kidneys for the toxicity evaluation. One representative mouse was picked from each group 24 hours after the fourth dosage, and tissues were investigated histologically after H&E staining. Magnification of lungs and kidneys was 400×, while liver was 100×.

As MMAE is highly toxic, we also studied the toxicities of TRAIL and its conjugates. The body weight of all groups kept stable during animal experiment except for MMAE group (Supplementary Figure S5). The average body weight of MMAE group decreased beneath 20 g at the end of experiment, indicating the existence of severe body damage caused by MMAE. Histological sections of the major organs from mouse were examined after H&E (Haematoxylin and eosin) staining (Fig. 6). It was found that lung bronchial epithelial cells (black arrow) were exfoliative and disordered, and pulmonary alveolus were full of erythrocytes. Besides, part of the hepatocytes were found to be apoptotic (Fig. 6, dashed arrow), suggesting severe liver toxicity. No obvious toxicities were found in other organs and groups. Therefore, the conjugation of MMAE with TRAIL or PEG-TRAIL could significantly reduce or even eliminate the toxicity resulted from MMAE.

## Discussion

Although TRAIL showed promising anticancer ability in preclinical studies, the shortcomings started to impede further development of TRAIL in clinical trials. Therefore, the drug delivery of TRAIL limited by poor pharmacokinetics, together with induced TRAIL resistance of tumor cells, probably were the major reasons of the withdrawing of TRAIL from Amgen and Genentech pipelines^14^. In order to improve the *in vivo* antitumor activities of TRAIL, these deficiencies should be addressed *via* strategies like fusion protein or protein modification^20^,^21^.

In present study, we hypothesize the site-specific hetero-modification of TRAIL trimer with PEG and MMAE at different monomers could improve the pharmacokinetics and pharmacodynamics of TRAIL at a time. Because PEG has been widely used to improve solubility and half-life of protein, while MMAE successfully conjugated with monoclonal antibody for tumor targeting therapy^11^,^13^,^22^. However, it is a tough challenge to co-conjugate TRAIL with PEG and MMAE efficiently and simply, meanwhile maintaining the apoptotic activity of TRAIL as much as possible. Previously, we reported the conjugation of TRAIL mutant with PEG or MMAE respectively for different purposes^9^,^14^. And the rapid conjugation was based on thioester bond formed between the mutated cysteine of TRAIL_95-281_ and maleimide group of MMAE or PEG, both of which improved the antitumor activity of TRAIL. Therefore, we also chose the TRAIL mutant N109C to co-conjugate with PEG and MMAE at a time. For the selective hetero-modification, we found the steric hindrance existed in the PEGylation of N109C, resulting one monomer left unPEGylated while all the three monomers were modified at the free N-terminus. As a result, we used a strategy that conjugate part of TRAIL monomers with PEG first, then the unmodified monomer continued to react with MMAE to achieve the hetero-modification of TRAIL trimer.

As depicted in Fig. 2a, the steric effects did exist during the co-modification, showing different products resulted from various reaction orders. Intriguingly, if TRAIL mutant N109C conjugate with PEG only 30 seconds ahead of MMAE, the hetero-modification could be accomplished. There might be an explanation that it took no more than 30 seconds for PEG to reach and “occupy” one or two of the reduced cysteines. According to the size-exclusion chromatography and Circular dichroism (CD) spectroscopy analysis results, the PEG-TRAIL-vcMMAE conjugates (PEG-N109C-vcMMAE) went through little alteration after two conjugation steps. And 1:1:1 PEG-N109C-vcMMAE conjugates would be a mixture of conjugates with different PEG/MMAE ratio (e.g., 0.5, 1 and 2), they could not be separated through size-exclusion chromatography, thus appearing as one peak. Therefore, these findings demonstrate our strategy of hetero-modification of TRAIL trimer was viable through taking advantage of steric effects, and the conjugation occurred at the potential N-glycosylation site (Asn-109 to Cys) met our expectations. What’s more, the first PEGylation step stabilized the TRAIL intermediates, and further decreased the aggregation resulted from the reaction between TRAIL and vcMMAE. Therefore, the yield of PEG-N109C-vcMMAE (~60%) was largely improved in comparison with N109C-vcMMAE (~25%).

PEG-N109C-vcMMAE showed attenuated apoptosis-inducing activity comparing with TRAIL or N109C-vcMMAE as expected, since additional PEGylation would affect the binding of TRAIL or its conjugates with death receptors. However, PEG-N109C-vcMMAE and N109C-vcMMAE other than TRAIL exerted cell cycle arrest capability conveyed from the conjugated MMAE, and PEG still had an impact on the behaviors of PEG-N109C-vcMMAE at cellular level, leading to its decreased MMAE release within tumor cell. Although the *in vitro* biological activities of PEG-N109C-vcMMAE were weakened because of co-modification, it has much superior bioavailability and half-life than TRAIL and N109C-vcMMAE, probably serving as a compensation of overall antitumor activity. Unexpectedly, the conjugation with MMAE also multiplied the half-life of TRAIL, which was further tremendously prolonged by the hetero-modification with PEG and MMAE. The reduced glomerular filtration of PEG-N109C-vcMMAE might be the major reason of improved pharmacokinetics due to the increased hydrodynamic radius, as a result of the hetero-modification.

Since molecular weight of MMAE is similar with sulfo-cyanine5-maleimide (Cy5), the PEG-N109C-Cy5 could mimic the *in vivo* targeting behaviours of PEG-N109C-vcMMAE. And non-binding control BSA-Cy5 were found to be systemically distributed in xenograft mouse model, the drug delivery by PEG-N109C-vcMMAE was revealed to be active tumor targeting, independent of EPR effect. Together with improved pharmacokinetics and delivery of highly toxic MMAE, active tumor targeting conferred the best *in vivo* antitumor activities to PEG-N109C-vcMMAE. Interestingly, according to the *in vitro* antitumor activities and TUNEL results of TRAIL, TRAIL should be the best antitumor agent instead of PEG-N109C-vcMMAE. It was obvious that TRAIL resistance was induced in TRAIL-sensitive NCI-H460 cells by repeated dosages of TRAIL. And this TRAIL resistance could be overcome via extra killing by free MMAE, and the non-charged MMAE could efflux outside of target tumor cells and keep attacking neighboring cancer cells (“bystander killing”)^23^. And the active tumor targeting of PEG-N109C-vcMMAE could also be confirmed by the performance of BSA-vcMMAE. Hence, although PEG-N109C-vcMMAE showed limited apoptosis-inducing ability *in vitro*, as a result of hetero-modification with PEG and MMAE, it was proved to be most potent during *in vivo* tumor growth inhibition. This result is more likely benefited from the multiplied half-life of PEG-N109C-vcMMAE after hetero-modification.

The toxicity study demonstrated that MMAE alone was highly toxic to lung and liver cells *in vivo*, while the conjugation with N109C or PEG-N109C could significantly reduce the systematic toxicity of MMAE. Erythrocytes flooded into pulmonary alveolus might be a consequence of exfoliated lung bronchial epithelial cells resulting from MMAE treatment. These findings demonstrate that PEG-N109C-vcMMAE and N109C-vcMMAE could specifically deliver MMAE to target tumor tissue, where the internalized and accumulated MMAE started killing TRAIL-resistant and -sensitive tumor cells. Besides, the escaped MMAE into normal tissues was not enough to exert side effects.

In summary, we have presented a novel and simple method for the co- and hetero-modification of TRAIL with PEG and MMAE at the same site from different monomers, and demonstrated the application of PEG-TRAIL-vcMMAE conjugates was a viable therapeutic strategy. PEG-TRAIL-vcMMAE conjugates were successfully synthesized taking advantage of steric effects during reaction. PEG-TRAIL-vcMMAE exhibited highly specific targeting capability, suggesting it has potential to deliver various drugs for different purposes. The favorable *in vivo* antitumor activities and improved half-life of PEG-TRAIL-vcMMAE along with no observed toxicity were powerful evidences of addressing the shortcomings of TRAIL. Further studies will focus on the effects of different PEG/MMAE modification ratios on cellular pharmacokinetics and *in vivo* antitumor activities of different PEG-TRAIL-vcMMAE conjugates.

## Methods

### Materials

All reagents were purchased from Sigma Aldrich unless otherwise noted. Milli-Q water (Millipore) was used throughout the experiment. The maleimidocaproyl-valine-citrulline-monomethylauristatin E (Mal-Val-Cit-MMAE) was prepared by Dr. David Miao as previously described^24^,^25^,^26^. Methoxy-PEG-maleimide (mPEG-MAL, 5000 Da) was purchased from SINOPEG (Xiamen, China). TRAIL_114-281_ and TRAIL_95-281_ mutant N109C were expressed and purified as previously reported^9^.

### Animal ethics

All animal experiments were carried out in accordance with the National Institute of Health Guide for the Care and Use of Laboratory Animals. The protocols were approved by the Committee on the Ethics of Animal Experiments of the Zhejiang University, China (SCXK 2007–0029).

### Preparation and characterization of TRAIL-vcMMAE and PEG-TRAIL-vcMMAE conjugates

TRAIL_114-281_, TRAIL_95-281_, TRAIL_95-281_ mutant N109C, TRAIL-vcMMAE and BSA-vcMMAE conjugates were prepared according to the method previously reported^9^. For the synthesis of PEG-TRAIL-vcMMAE conjugates, the TRAIL mutant N109C (1 mg/mL) in PBS (pH 7.4) was treated with Tris (2-Carboxyethyl) phosphine Hydrochloride (TCEP HCl) (10 eq) at 37 °C for 1 h to disrupt intermolecular disulfide bond. To the reduced N109C at room temperature was added the methoxyl-PEG-maleimide (mPEG-MAL, 5000 Da) (2 eq/SH group) in PBS, or vcMMAE (4 eq/SH group) in CH_3_CN (30% v/v). After 40 min, vcMMAE (4 eq/SH group) or mPEG-MAL (2 eq/SH group) was added to the above reactions for the co-modification of TRAIL mutant. The mPEG-MAL (2 eq/SH group) was added 30 seconds ahead of vcMMAE (4 eq/SH group) as the third condition. All the conjugates were analyzed by 12% SDS-PAGE. For the subsequent *in vitro* and *in vivo* expriments, TRAIL-vcMMAE and PEG-TRAIL-vcMMAE conjugates were desalted by centrifugal ultrafiltration (Amicon Ultra-0.5 mL 10 K, Millipore), and then sterile filtered. Protein and drug concentrations were determined by spectral analysis. Circular dichroism (CD) spectra were recorded on a Chirascan Plus CD spectrophotometer (Applied Photophysics Co., Ltd). TRAIL_95-281_, TRAIL-vcMMAE and PEG-TRAIL-vcMMAE conjugates were diluted in PBS (pH 7.4) to a final concentration of 1 mg/mL for far-UV CD measurements. CD spectra were obtained with a bandwidth of 1 nm and time-per-potint of 1s (25 μs × 40,000). Pathlength: 0.2 mm. Wavelength: 195 nm−260 nm. Scans were performed three times.

### Cell culture

K562 human erythromyeloblastoid leukemia cell line, MCF-7 human breast cancer cell line and NCI-H460 human large cell lung carcinoma cell line were obtained from the Cell bank of the Chinese Academy of Sciences (Shanghai) and were cultured in RPMI-1640 medium (K562 and NCI-H460 cells) or DMEM/F-12 medium (MCF-7 cells) (Gibco, life technologies), supplemented with 10% fetal bovine serum (FBS) (Gibco, life technologies), 100 U/mL of penicillin, and 100 μg/mL of streptomycin, at 37 °C under a 5% CO_2_ atmosphere.

### Studies on apoptotic mechanism of PEG-N109C-vcMMAE conjugates

#### (1) Study on the internalization mechanism of conjugates

NCI-H460 or MCF-7 cells were seeded at a density of 2 × 10^4^ cells/mL in 96-well plates. After incubated for 24 h, cells were treated with TRAIL_114-281_ (40 ng/mL) or PEG-N109C-vcMMAE conjugates (100 g/mL or 200 ng/mL) plus serial concentration DR4-Fc/DR5-Fc (0–400 ng/mL) for 1 (TRAIL_114-281_) or 4 days (PEG-N109C-vcMMAE). Cell viability was determined by Cell Counting Kit-8 (Dojindo, Osaka, Japan). The absorbance of 450 nm was measured by BioRad Model 680 Microplate Reader. The results were expressed as mean  ± standard deviation (S.D.) (n = 3).

#### (2) Blockage of the extrinsic (TRAIL) pathway *via* the pan-caspase inhibitor Z-VAD-FMK

NCI-H460 cells were seeded at a density of 2 × 10^6^ cells/mL (3 mL) in 6-well plates. After incubated for 24 h, cells in control groups were treated with TRAIL_114-281_ (20 ng/mL), MMAE (20 ng/mL) for 24 h or PEG-N109C-vcMMAE conjugates (100 ng/mL) for 72 h. On the other hand, cells in experimental groups were treated as above except for the addition of 10 μM Z-VAD-FMN (Selleck chemicals, United states). After staining with Annexin V-FITC Apoptosis Detection Kit (Beyotime, China), the apoptosis of cell samples were examined by flow cytometry (FC500MCL, Beckman).

### Selective induction of G_2_ growth arrest

MCF-7 cells were incubated with 1 μM N109C, N109C-vcMMAE, PEG-N109C-vcMMAE and 0.5 μM MMAE for 8 h. After exposure, cells were collected, fixed with ice-cold ethonal (70% v/v) for 24 h, then washed with PBS (pH 7.4) twice and stained with propidium iodide (PI) for 30 min in the dark. After PI staining, cells were examined by flow cytometry (FC500MCL, Beckman).

### *In vivo* pharmacokinetics of TRAIL mutant N109C, N109C-vcMMAE and PEG-N109C-vcMMAE

Twelve Sprague-Dawley (SD) rats (male, 170 ~ 200 g) were divided randomly into 3 groups (n = 4). Each rat in the experimental group was injected (i.v.) via tail vein with 1 mg TRAIL_95-281_ mutant N109C, N109C-vcMMAE or PEG-N109C-vcMMAE (~5 mg/kg), and the control group was injected with PBS (pH 7.4). Blood samples were obtained at different time points after administration, and then were incubated at 37 °C for 30 min followed by centrifugation (3000 rpm, 20 min) to collect serum. The concentration of N109C protein and its conjugates in serum were determined by direct Enzyme-linked immunosorbent assay (ELISA) according to a previous protocol^20^. Briefly, N109C, N109C-vcMMAE and PEG-N109C-vcMMAE standard were diluted to serial concentrations by coating buffer, and the linear range of the sigmoidal titration curve was used to calculate the concentrations of N109C and its conjugates in rat blood. Serum samples were diluted to several final concentrations to locate their concentrations in the linear range. Pharmacokinetic parameters were obtained from serum TRAIL concentration profiles using non-compartment model analysis in DAS 2.0 software (China). Aside from the conventional ELISA method for the quantification of TRAIL or mutants, antibody-free LC-MS/MS quantification of TRAIL was also developed^27^.

### *In vivo* distribution of PEG-N109C-Cy5

#### (1) Synthesis of BSA-Cy5 and PEG-N109C-Cy5

For the synthesis of PEG-TRAIL-Cy5 conjugates, the TRAIL mutant N109C (1 mg/mL) in PBS (pH 7.4) was treated with TCEP HCl (10 eq) at 37 °C for 1 h to disrupt intermolecular disulfide bond. To the reduced N109C at room temperature was added the methoxyl-PEG-maleimide (mPEG-MAL, 5000 Da) (2 eq/SH group) in PBS. After 40 min, sulfo-cyanine5-maleimide (water-soluble Cy5, a near- IR fluorophore) (4 eq/SH group) dissolved in PBS was added to the above reactions for the co-modification of TRAIL mutant with PEG and Cy5. For the preparation of BSA-Cy5, BSA (1 mg/mL) was treated with TCEP HCl (10 eq) at 37 °C for 1 h to disrupt intermolecular disulfide bond. To the reduced BSA at room temperature was added sulfo-cyanine5-maleimide (2 eq/SH group), and reacted for 40 min. All the reactions were kept from light.

For the subsequent *in vivo* distribution expriments, BSA-Cy5 and PEG-N109C-Cy5 conjugates were desalted by centrifugal ultrafiltration (Amicon Ultra-0.5 mL 10K, Millipore), and then sterile filtered. Protein and drug concentrations were determined by spectral analysis. Due to the similar molecular weight of Cy5 (803 g/mol) with vcMMAE (1316.63 g/mol), Cy5-maleimide could be an ideal fluorescent marker without deviating from structural effect of conjugating PEG-N109C with MMAE.

#### (2) *In vivo* distribution of PEG-N109C-Cy5 and BSA-Cy5

Athymic nude mice (Slaccas Laboratory Animal Co., Ltd., Shanghai, China) were used for all *in vivo* experiments. To build the tumor-bearing mice model, freshly harvested NCI-H460 cells (5 × 10^6^ cells per mouse) were inoculated subcutaneously in the right or both flanks of athymic nude mice. PEG-N109C-Cy5 and BSA-Cy5 were injected intravenously into NCI-H460 tumor-bearing athymic nude mice via tail vein (~20 μg, Cy5 based). BSA-Cy5 was used as a non-binding control. The mice were monitored for the fluorescence at 30 min and every 24h using the Maestro *in-vivo* imaging system (CRi, USA). At 96 h, the mice were sacrificed and the organs (tumor, liver, heart, lung, kidney, spleen) were harvested and imaged immediately. The filter set (orange: excitation, 586 nm ~601 nm; emission, 635-nm long pass) was used to detect Cy5 fluorescence. Fluorescent and photographic images were acquired and overlaid. The pseudocolor image represents the spatial distribution of BSA-Cy5 and PEG-N109C-Cy5 in the organs. Background fluorescence was subtracted before analysis.

### *In vivo* antitumor activities of TRAIL and its conjugates

Balb/c athymic nude mice (Slaccas Laboratory Animal Co., Ltd., Shanghai, China) were used for *in vivo* antitumor activities of TRAIL and its conjugates. Fourty-four athymic nude mice (female, 6–8 weeks) were divided into 7 groups [saline (6 mice), MMAE (390 μg/kg) (4 mice), BSA-vcMMAE (10 mg/kg, 390 μg/kg MMAE) (3 mice), TRAIL_114-281_ 10 mg/kg (4 mice), PEG-N109C-vcMMAE (4 mg/kg, 65 μg/kg MMAE) (5 mice), PEG-N109C-vcMMAE (24 mg/kg, 390 μg/kg MMAE) (6 mice), N109C-vcMMAE (10 mg/kg, 390 μg/kg MMAE) (6 mice)].To build the tumor-bearing mice model, freshly harvested NCI-H460 cells (5 × 10^6^ cells per mouse) were inoculated subcutaneously in the right flank of athymic nude mice. When tumor volume reached 50 mm^3^, mice were treated with above samples once every two days for four times (q2d × 4) intravenously. Tumor volumes were continuously monitored until the end of the experiment and calculated by the formula V = (L × W^2^)/2, L and W refer to longitudinal and transverse tumor diameters respectively. The body weight of mice was also monitored once every two days .

*In vivo* cell apoptosis was evaluated by terminal deoxynucleotidyl transferase–mediated dUTP nick end labeling (TUNEL) assays (*In Situ* Cell Death Detection kit, Roche). The tumor tissues for TUNEL assay were obtained from 6 groups (except BSA-vcMMAE group) above randomly (one mouse per group), 24 h after the fourth administration of samples. After fixed with 10% neutral-buffered formalin and paraffin-embedded, sections (5 μm) were cut from tumor tissues, developed by TUNEL reagents after route dewaxing and washing steps and imaged by a light microscope. Lung, Liver and kidney tissues were also obtained from the same mice and immediately fixed by 10% neutral-buffered formalin for the following toxicity evaluation.

Acute liver or kidney toxicity was studied through H&E (Hematoxylin and Eosin) staining for the morphological examination of tissue cells. In brief, the lung, liver and kidney tissues obtained were fixed, paraffin-embedded and stained by H&E according to above-mentioned steps. The sections were examined by light microscopes at the magnification 400× (lung and kidney) or 100× (liver).

### Statistical analysis

One-way and two-way analysis of variance (ANOVA) were used to determine statistical significance (*P < 0.05, **P < 0.01 and ***P < 0.001).

## Additional Information

**How to cite this article**: Pan, L.-Q. *et al*. Hetero-modification of TRAIL trimer for improved drug delivery and *in vivo* antitumor activities. *Sci. Rep*. **5**, 14872; doi: 10.1038/srep14872 (2015).

## Supplementary Material

Supplementary Information

## Figures and Tables

**Figure 1:**
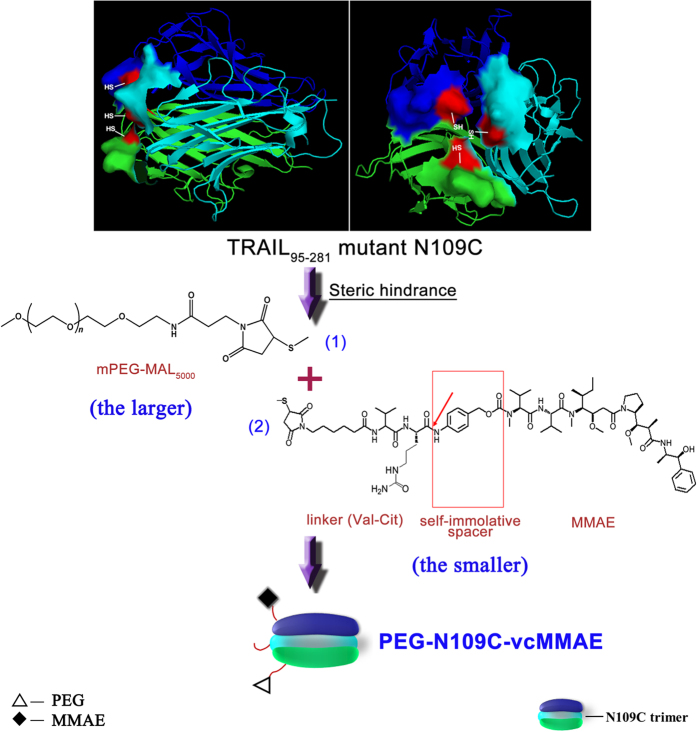
Strategy of the co-modification of TRAIL mutant with PEG and MMAE. PEG refers to methoxy- polyethylene glycol -maleimide (mPEG-MAL, 5000 Da). Monomethyl Auristatin E is abbreviated as MMAE, and linker (Val-Cit) represents maleimidocaproyl-valine-citrulline; Self-immolative spacer refers to *p*-aminobenzylcarbamate. N109C represents the Asn-109 to Cys mutation of TRAIL (95–281 amino acids). The arrow indicates the Cathepsin B cleavage site of the linker.

**Table 1:** Comparison of pharmacokinetic parameters after administering N109C, N109C-vcMMAE and PEG-N109C-vcMMAE to rats at 1 mg/rat i.v. (5 mg/kg) (n = 4).

Pharmacokineticparameters	N109C	N109C-vcMMAE	PEG-N109C-vcMMAE
AUC_inf_^1^ (mg/L*h)	178.16 ± 80.18	756.63 ± 368.49	1917.52 ± 555.72
t_1/2_^2^ (h)	1.91 ± 0.94	4.26 ± 3.34	11.54 ± 6.20
CL^3^ (L/h/kg)	0.034 ± 0.017	0.0070 ± 0.0030	0.0050 ± 0.0010

^1^AUCinf: Area under the curve from zero to infinity.

^2^t_1/2_: Half-life.

^3^CL: Clearance.
