# Supplementary material for: Hetero-modification of TRAIL trimer for improved drug delivery and in vivo antitumor activities
Source: Sci Rep. 2015 Oct 8;5:14872. doi: 10.1038/srep14872 (PMC4597189; doi:10.1038/srep14872)
Supplement: Supplementary Information [file srep14872-s1.pdf]

Supporting Information

Hetero-modification of TRAIL trimer for improved drug delivery and in vivo antitumor activities

Li-Qiang Pan[1], *Wen-Bin Zhao*[1], *Jun Lai*[1], *Ding Ding*[2], *Xiao-Yue Wei*[1], *Yang-Yang Li*[1], *Wen-Hui Liu*[1], *Xiao-Yue Yang*[1], *Ying-Chun Xu*[1], *and Shu-Qing Chen*[1]*

1. Department of Drug metabolism and Drug analysis, College of Pharmaceutical Sciences, Zhejiang University, Hangzhou, 310058, China
E-mail: chenshuqing@zju.edu.cn

2. Zhejiang Hisun Pharmaceutical Inc, 46 Waisha Road, Jiaojiang District, 317000 Taizhou, China

Keywords: Drug delivery, TRAIL conjugates, TRAIL resistance, Monomethyl Auristatin E, antitumor agents

<u>Preparation of TRAIL-vcMMAE and PEG-TRAIL-vcMMAE conjugates.</u>

Steps:

Number	Reduction	First Step	Second step
1	TCEP (10 eq)<br>1 h, 37 ℃	mPEG-MAL<br>(2 eq/ SH group)<br>40 min	vcMMAE<br>(4 eq / SH group)<br>40 min
2		vcMMAE<br>(4 eq / SH group)<br>40 min	mPEG-MAL<br>(2 eq/ SH group)<br>40 min
3		mPEG-MAL was added 30 seconds ahead of vcMMAE, and incubated together for 40 min at RT	

<u>Characterization of N109C-vcMMAE</u>

<u>1) Q-TOF MS (mass spectrometry)</u>

$TRAIL_{114-281}$, N109C and N109C-vcMMAE were diluted by dd H_2O to a final concentration of 1 mg/mL, and centrifuged (12,000 rpm, 15 min). Supernatant was analyzed by Q-TOF MS.

Instrument: Waters UPLC Acquity Bio H Class, Waters Xevo G2-S Q TOF

Column：MassPREP Desalting column

Column temp: 80°C

A: 0.1% Formic Acid in H_2O

B: 0.1% Formic Acid in ACN

Gradient:

Time(min)	Flow Rate(mL/min)	%A	%B
0	0.200	90.0	10.0
2.00	0.200	90.0	10.0
13.00	0.200	20.0	80.0
13.50	0.200	10.0	90.0
15.50	0.200	10.0	90.0
16.00	0.200	90.0	10.0
18.00	0.200	90.0	10.0

MS parameters:

Capillary (kV): 2.5

Sampling Cone (V): 60

Source Temperature (°C): 100

Desolvation Temperature (°C): 500

Desolvation Gas Flow (L/Hr): 800.0

Mass range(m/z): 400 to 4,000

Results:

Subject	Expected MW (Da)	Calculated MW (Da)
$TRAIL_{114-281}$ monomer	19491.85	19497.97
N109C monomer	21652.23	21657.97
N109C dimer	43302.46	43316.8
N109C-vcMMAE monomer	22966.86	22969.8

Due to the high column temperature, all the trimeric TRAIL and its conjugates were depolymerized in monomers (Figure S1). And because PEG is mixture of polymers with different molecular weight (MW), so we did not determine its MW through Q-TOF MS. These results indicated that all the proteins were expressed in *E.coli* correctly, and TRAIL mutant N109C was successfully conjugated with MMAE.

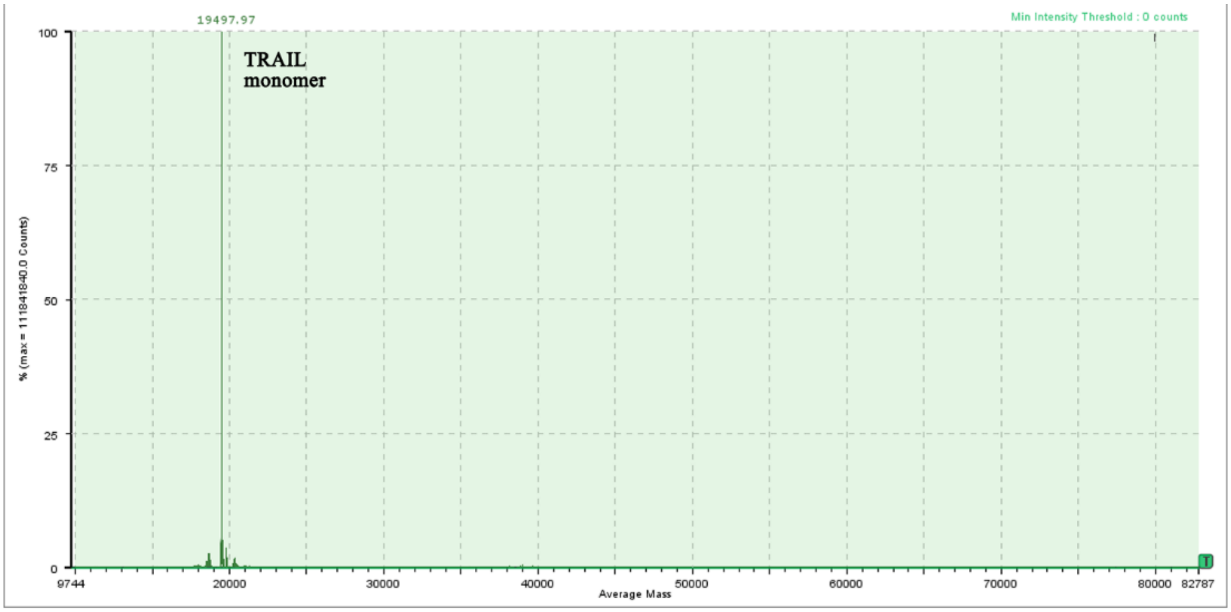
19497.97
TRAIL monomer
Min Intensity Threshold : 0 counts
% (max = 111841840.0 Counts)
100
75
50
25
0
9744
20000
30000
40000
50000
60000
70000
80000
82787
Average Mass

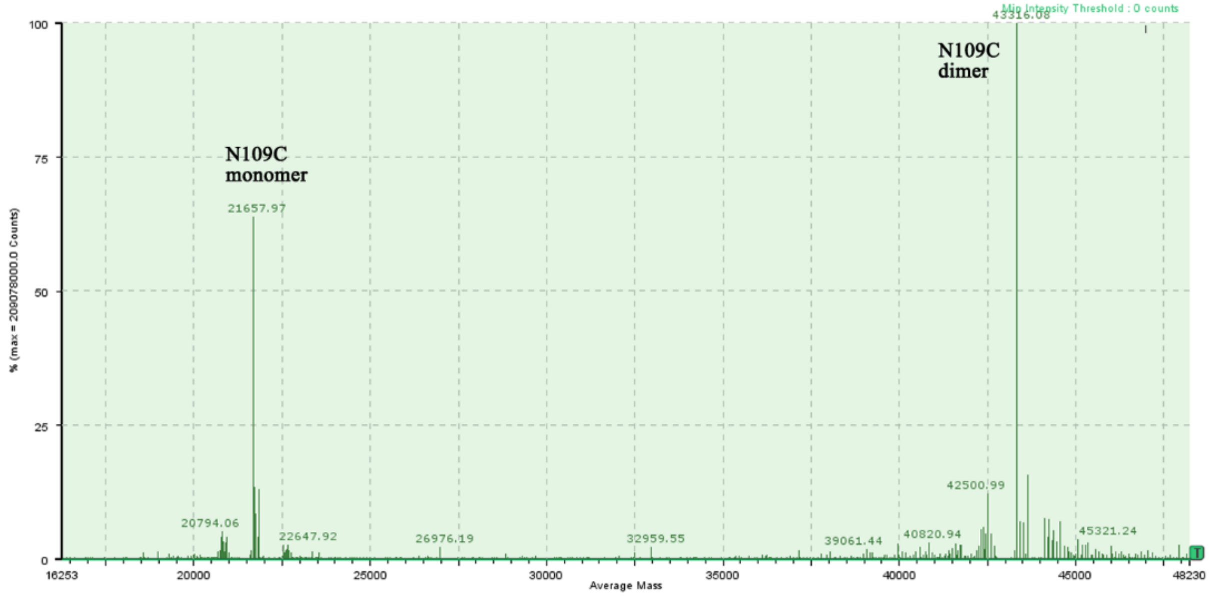
Min Intensity Threshold : 0 counts
43316.08
N109C dimer
N109C monomer
21657.97
20794.06
22647.92
26976.19
32959.55
39061.44
40820.94
42500.99
45321.24
% (max = 209078000.0 Counts)
100
75
50
25
0
16253
20000
25000
30000
35000
40000
45000
48230
Average Mass

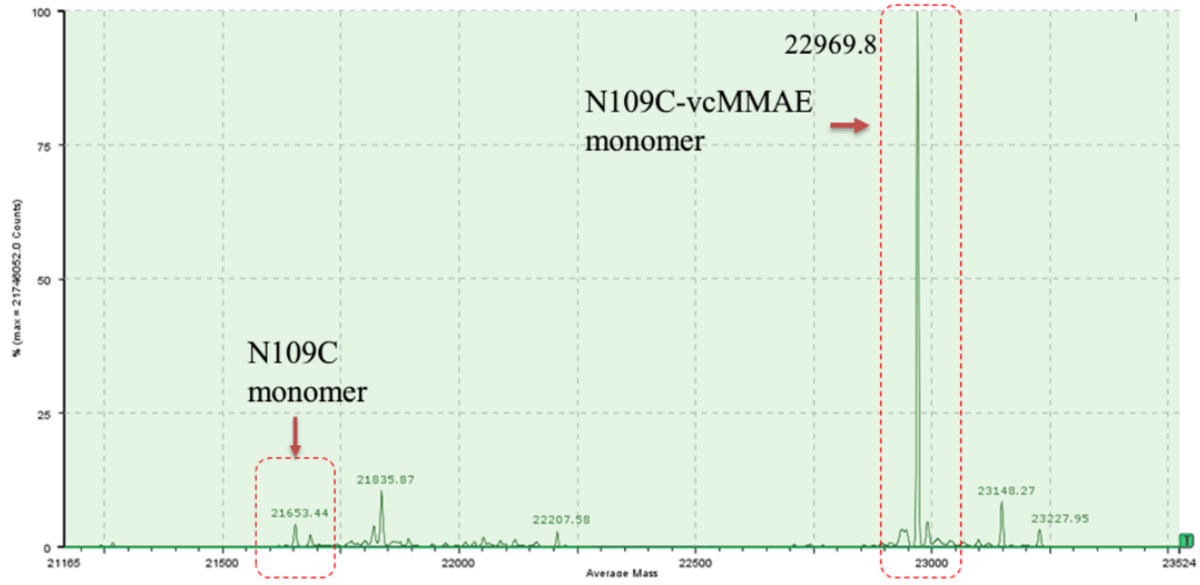
22969.8
N109C-vcMMAE monomer
N109C monomer
21653.44
21835.87
22207.58
23148.27
23227.95
% (max = 217460052.0 Counts)
100
75
50
25
0
21165
21500
22000
22500
23000
23524
Average Mass

Figure S1. Q-TOF MS results of $TRAIL_{114\text{-}281}$, N109C and N109C-vcMMAE

1) <u>Size-exclusion chromatography (SEC)</u>

A silica-based G3000SW_{XL} size exclusion column (7.8 mm×30 cm dimension, 5 µm particle size, 250 Å pore size) (Tosoh Bioscience LLC, Montgomeryville, PA, USA) was used to determine the polymerization of N109C and its conjugates. The separation was performed using size exclusion column with mobile phase (50 mM NaH_2PO_4 (pH 6.7), 300 mM NaCl, 0.05% NaN_3) at flow rate of 0.6 mL/min. The injection volume was 20 µL.

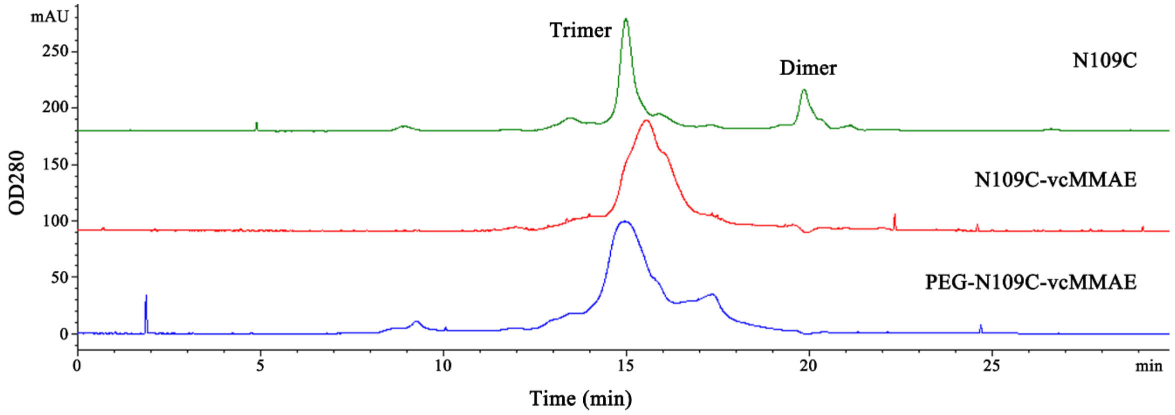

Figure S2. Size-exclusion chromatography analysis of the tertiary structure of N109C and its conjugates.

***In vitro* antitumor activity of PEG-TRAIL-vcMMAE conjugates**

NCI-H460 is human large cell lung cancer cell line (TRAIL sensitive); K562 is human erythromyeloblastoid leukemia cell line (TRAIL resistant); MCF-7 is human breast adenocarcinoma cell line (TRAIL resistant). All tumor cells were seeded at a density of 3×10^3 cells/100 µL/well in 96-well microtiter plates (Costar, Corning). Twenty-four hours post-seeding, the cultures were washed twice with PBS and then exposed to various concentrations (1.6~250 nM) of $TRAIL_{114\text{-}281}$, N109C-vcMMAE and PEG-N109C-vcMMAE for 96 h. The PBS (pH 7.4) was used as negative control. Cell viability was determined by Cell Counting Kit-8 (Dojindo, Osaka, Japan). The absorbance of 450 nm was measured by BioRad Model 680 Microplate Reader. The results were expressed as mean ±standard deviation (S.D.) (n =3).

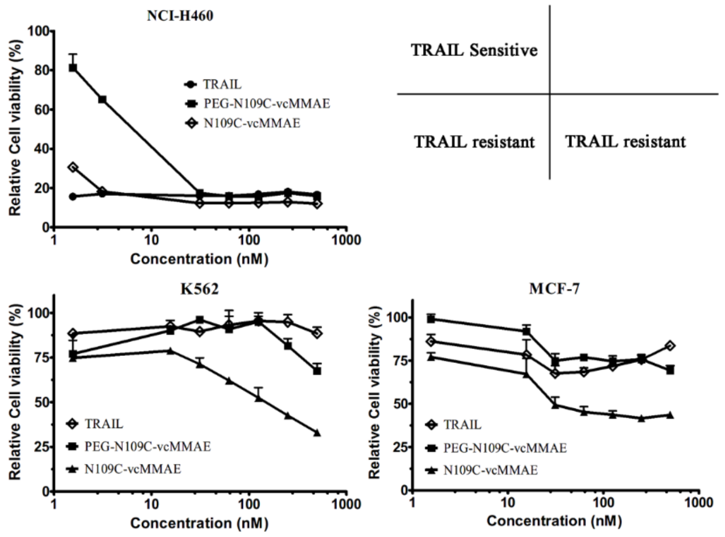

Figure S3. In vitro antitumor activities of TRAIL and its conjugates on differents tumor cell lines.

In vivo antitumor activities of TRAIL and its conjugates

Balb/c athymic nude mice (Slaccas Laboratory Animal Co., Ltd., Shanghai, China) were used for in vivo antitumor activities of TRAIL and its conjugates. Fourty-four athymic nude mice (female, 6-8 weeks) were divided into 7 groups [saline (**6 mice**), MMAE (390 μg/kg) (**4 mice**), BSA-vcMMAE (10 mg/kg, 390 μg/kg MMAE) (**3 mice**), $TRAIL_{114-281}$ 10 mg/kg (**4 mice**), PEG-N109C-vcMMAE (4mg/kg, 65 μg/kg MMAE) (**5 mice**), PEG-N109C-vcMMAE (24mg/kg, 390 μg/kg MMAE) (**6 mice**), N109C-vcMMAE (10 mg/kg, 390 μg/kg MMAE) (**6 mice**)].To build the tumor-bearing mice model, freshly harvested NCI-H460 cells (5×10^6 cells per mouse) were inoculated subcutaneously in the right flank of athymic nude mice. When tumor volume reached 50 mm^3, mice were treated with above samples once every two days for four times (q2d×4) intravenously. Tumor volumes were continuously monitored until the end of the experiment and calculated by the formula $V = (L*W^2)/2$, L and W refer to longitudinal and transverse tumor diameters respectively. The body weight of mice was also monitored once every two days (**Figure S4**).

In vivo cell apoptosis was evaluated by terminal deoxynucleotidyl transferase–mediated dUTP nick end labeling (TUNEL) assays (In Situ Cell Death Detection kit, Roche). The tumor tissues for TUNEL assay were obtained from 6 groups (except BSA-vcMMAE group) above randomly (one mouse per group), 24 h after the fourth administration of samples. After fixed with 10% neutral-buffered formalin and paraffin-embedded, sections (5 μm) were cut from tumor tissues, developed by TUNEL reagents after route dewaxing and washing steps and imaged by a light

microscope(**Figure S5**). Lung, Liver and kidney tissues were also obtained from the same mice and immediately fixed by 10% neutral-buffered formalin for the following toxicity evaluation. Acute liver or kidney toxicity was studied through H&E (Hematoxylin and Eosin) staining for the morphological examination of tissue cells. In brief, the lung, liver and kidney tissues obtained were fixed, paraffin-embedded and stained by H&E according to above-mentioned steps. The sections were examined by light microscopes at the magnification 400× (lung and kidney) or 100× (liver).

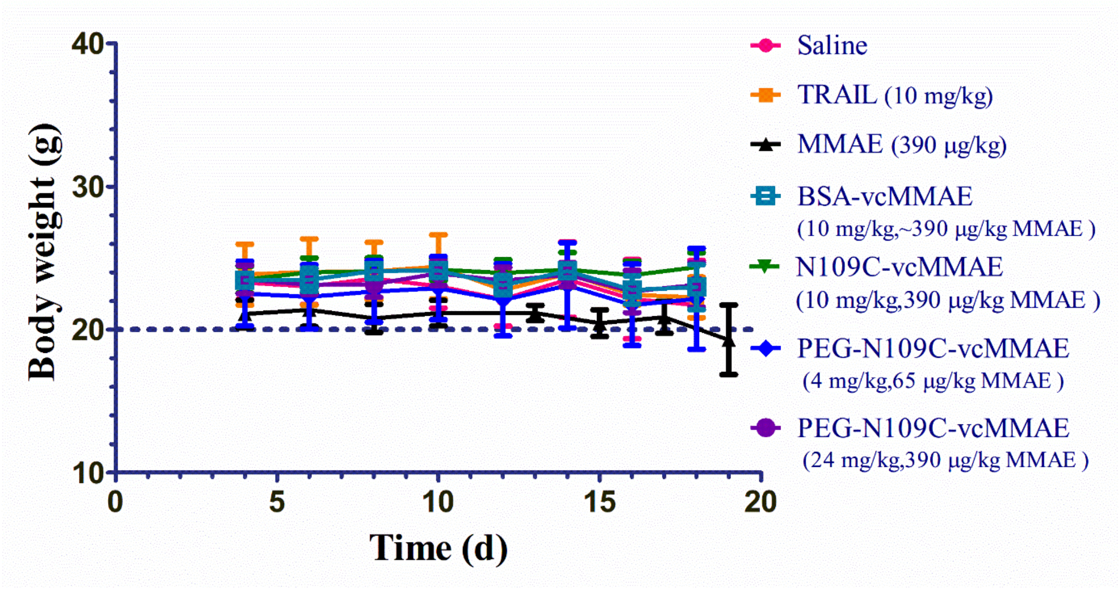

Figure S4. Body weight monitoring of mice treated with different sampls.

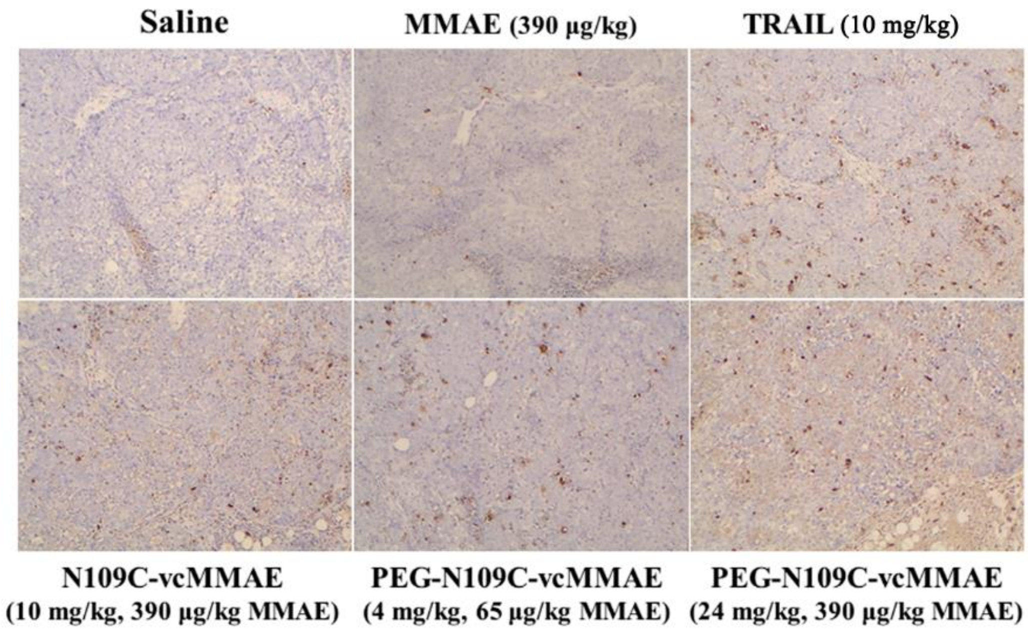

Figure S5. TUNEL assays for the in situ cell death within tumor tissues. Magnification 100×.
